# Highly Variable Genomic Landscape of Endogenous Retroviruses in the C57BL/6J Inbred Strain, Depending on Individual Mouse, Gender, Organ Type, and Organ Location

**DOI:** 10.1155/2017/3152410

**Published:** 2017-08-29

**Authors:** Kang-Hoon Lee, Debora Lim, David Greenhalgh, Kiho Cho

**Affiliations:** ^1^Department of Surgery, University of California, Davis, Sacramento, CA, USA; ^2^Shriners Hospitals for Children Northern California, Sacramento, CA, USA

## Abstract

Transposable repetitive elements, named the “TREome,” represent ~40% of the mouse genome. We postulate that the germ line genome undergoes temporal and spatial diversification into somatic genomes in conjunction with the TREome activity. C57BL/6J inbred mice were subjected to genomic landscape analyses using a TREome probe from murine leukemia virus-type endogenous retroviruses (MLV-ERVs). None shared the same MLV-ERV landscape within each comparison group: (1) sperm and 18 tissues from one mouse, (2) six brain compartments from two females, (3) spleen and thymus samples from four age groups, (4) three spatial tissue sets from two females, and (5) kidney and liver samples from three females and three males. Interestingly, males had more genomic MLV-ERV copies than females; moreover, only in the males, the kidneys had higher MLV-ERV copies than the livers. Perhaps, the mouse-, gender-, and tissue/cell-dependent MLV-ERV landscapes are linked to the individual-specific and dynamic phenotypes of the C57BL/6J inbred population.

## 1. Introduction

The vast majority of the core concepts and relevant methodologies for modern studies of normal and disease biology are typically tethered to the function and polymorphism of “conventional” genes. Conventional gene sequences are reported to be shared, with a homology of greater than 80%, among a wide range of species, ranging from rodents to humans [1, 2]. A survey of the data obtained from recent biomedical investigations, which focus on the function and polymorphism of conventional genes, indicates that the ratio of tangible and/or helpful returns is very low, in consideration of the enormous investments [3–7].

The announcements in 2001 and 2002 that the human and mouse reference genomes, respectively, were “completely” sequenced were followed by numerous publications which reported “whole” genome sequences of a wide range of species [1, 8–13]. These whole genome projects were apparently executed on a platform of a static genome within an individual [14–16]. However, it is interesting to note that not even a single chromosome of the human and mouse reference genomes, not to mention the other mammalian genomes, has been fully decoded as of 2016 (National Center for Biotechnology Information [NCBI], National Institutes of Health). For instance, less than half of human chromosome Y has been decoded.

According to the current annotation information from major human and mouse genome databases, the sum of exons pertaining to the conventional genes is estimated to occupy ~1.2% of the presumed full-length genomes [1, 11]. On the other hand, transposable repetitive elements, named the “TREome” in this study, are expected to comprise ~46% and ~38.6% of the human and mouse genomes, respectively [1]. There are four main TREome families: endogenous retroviruses (ERVs), long interspersed nuclear elements (LINEs), short interspersed nuclear elements (SINEs), and DNA transposons. Often, the transcripts of ERVs are referred to as “noncoding” long RNA species; however, it has been documented over the last few decades that some ERVs (mostly from mouse, pig, and human) are coded with *genes* capable of producing proteins with specific functions [17–19]. In addition, temporally and spatially acquired activities of the TREome are capable of altering the genome configuration with its “copy and paste” function (ERVs, LINEs, and SINEs) [20, 21].

Publications and databases, such as the NCBI human and mouse reference genomes, commonly define the sizes of the genomes/chromosomes of various species based on an assumption that their configurations are fully characterized and rather static [22, 23]. The finding that the length of mouse chromosome Y (~92 Mb) in NCBI's Annotation Release 105 reference genome (released in 2013) is almost six times larger than in the previous annotation of ~16 Mb in NCBI build 37.2 indicates that the current size estimates of genomes/chromosomes need to be reevaluated for each species (http://www.ncbi.nlm.nih.gov/projects/mapview/map_search.cgi?taxid=10090&build=105.0, http://www.ncbi.nlm.nih.gov/projects/mapview/map_search.cgi?taxid=10090&build=37.2) [24]. A recent report that the sizes and structures of the C57BL/6J inbred mouse genomes are temporally and spatially changed in conjunction with differential TREome activities suggests that (1) it is impractical to identify a single representative genome for an individual mouse or human from a pool of variant somatic genomes and (2) genome dynamics is linked to a range of biological processes, such as differentiation, stress response, and aging [25, 26].

In this study, we examined the extent of variations in the somatic genomes of the C57BL/6J inbred strain depending on individual mouse, gender, and space by using murine leukemia virus-type ERVs (MLV-ERVs) as a genome-wide TREome landscaping probe.

## 2. Materials and Methods

### 2.1. Animal Experiments

C57BL/6J inbred mice (females and males) of varying ages were purchased from the Jackson Laboratory (Bar Harbor, ME; West Sacramento, CA) or obtained from Dr. David Pleasure at the University of California, Davis (UC Davis). All animals were provided with water and food ad libitum during their housing at a UC Davis facility where some of them were aged for an extended period of time. The animal experiment protocol was approved by the Animal Use and Care Administrative Advisory Committee of UC Davis [27]. Animals were sacrificed by CO_2_ inhalation to collect sperm and/or tissues followed by snap-freezing in liquid nitrogen.

### 2.2. Genomic DNA Isolation and MLV-ERV Landscaping by Inverse-PCR (I-PCR) Analyses

**Figure 1:**
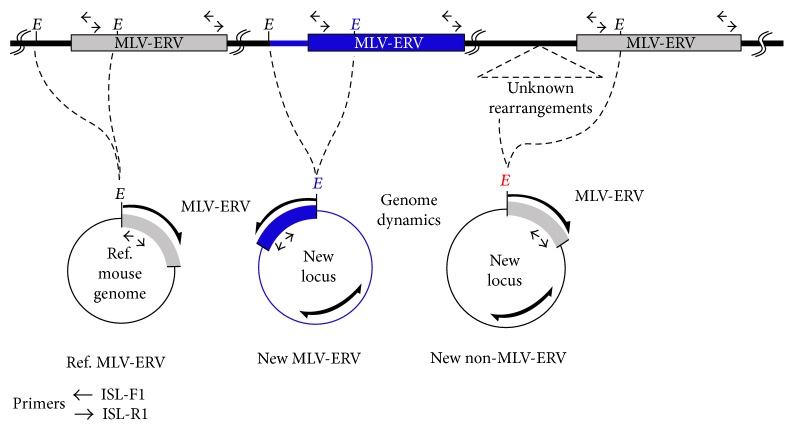
Illustration of the inverse-PCR (I-PCR) strategy for genomic MLV-ERV landscaping. The processes of I-PCR for MLV-ERV landscaping analyses of genomic DNA are illustrated. *E*: restriction enzyme recognition site; MLV-ERV: murine leukemia virus-type ERV; Ref.: reference; ISL-F1 and ISL-R1: primer names.

Snap-frozen sperm and somatic tissue samples were subjected to genomic DNA isolation using a DNeasy Tissue kit (Qiagen, Valencia, CA), and DNA samples were normalized to 20 ng/*μ*l. As an initial step for the I-PCR analyses (Figure 1()), genomic DNA (300 ng) was digested with NcoI (New England Biolabs, Ipswich, MA) at 37°C for 4 hours followed by self-ligation of the digests using T4 ligase (Promega, Madison, WI) overnight at 4°C. The MLV-ERV landscape information was collected by I-PCR amplification of the junctions spanning putative MLV-ERV integration loci using 2 *μ*l of the ligation products, Taq polymerase (Qiagen), and a pair of inverse primers (ISL-F1 and ISL-R1) designed from the conserved MLV-ERV sequences. The primer sequences and PCR conditions are listed in Supplementary Table 1 available online at https://doi.org/10.1155/2017/3152410. I-PCR products were resolved on a 7.5% polyacrylamide gel followed by ethidium bromide staining for visualization.

### 2.3. Real-Time Genomic DNA PCR Analyses of MLV-ERV Copy Numbers

For the kidney and liver genomic DNA isolated from six 19-month-old mice (3 females and 3 males), real-time PCR was performed using an MX3005P instrument (Agilent, Santa Clara, CA) with a reagent kit (Brilliant SYBR Green QPCR Master Mix) from Agilent and 25 ng of each genomic DNA in triplicate for each mouse. Details for the primers (ERV-U1 and ERV-U2) and PCR conditions are listed in Supplementary Table 1.

### 2.4. Copy Number Calculation and Statistical Analysis

The initial results from the real-time DNA PCR analyses of MLV-ERV copies were calculated as a relative copy number per single copy of the hypoxanthine phosphoribosyl transferase (HPRT) gene using a modified delta-delta CT method (2^(CT_(HPRT)_ − CT_(MLV − ERV)_)) [28]. A one-way ANOVA was used to determine the significance of differences in relative MLV-ERV copy number values between individual pairs of groups. Statistical significance was indicated when the *P* value was less than 0.05.

## 3. Results

### 3.1. Spatial Variations in the MLV-ERV Landscapes among Germ Line and Somatic Genomes in a Single C57BL/6J Inbred Mouse

**Figure 2:**
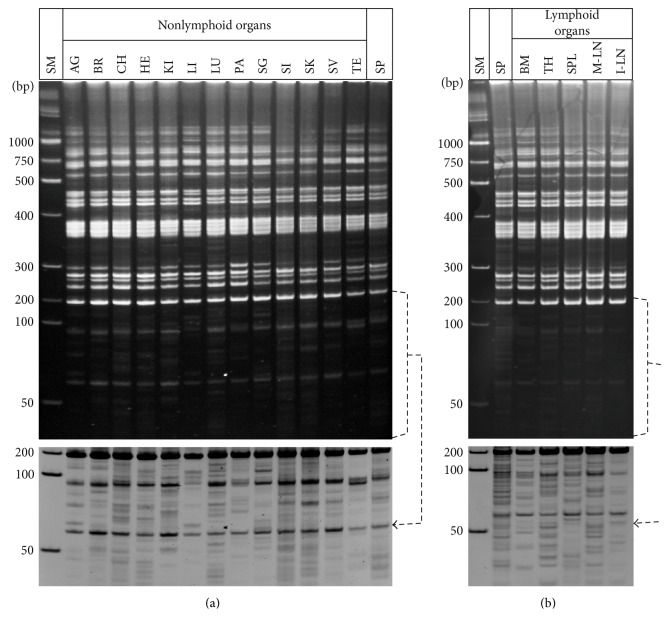
Spatial variations in the MLV-ERV landscapes among germ line and somatic genomes from a single C57BL/6J inbred mouse. (a) Variable MLV-ERV landscapes of 13 nonlymphoid organ and sperm genomic DNA samples. MLV-ERV landscapes were highly variable among the 13 nonlymphoid organ and sperm genomic DNA samples derived from a single C57BL/6J mouse although they share a collection of I-PCR amplicon bands. A reversed image of the selected area is presented for better resolution. SM: size marker; SP: sperm; AG: adrenal gland; BR: brain; CH: cerebellar hemisphere; HE: heart; KI: kidney; LI: liver; LU: lung; PA: pancreas; SG: salivary gland; SI: small intestine; SK: skin; SV: seminal vesicle; TE: testes. (b) Variable MLV-ERV landscapes of five lymphoid organ and sperm genomic DNA. Five lymphoid organ and sperm genomic DNA samples, derived from the same mouse as in (a), had variations in banding patterns of I-PCR amplicons. A reversed image of the selected area is presented for better visualization. SM: size marker; SP: sperm; BM: bone marrow; TH: thymus; SP: spleen; M-LN: mesenteric lymph node; I-LN: inguinal lymph node.

Current understanding of inbred mouse genetics projects that the genomic configuration is virtually identical within a population of mice from an inbred strain ([29], http://emice.nci.nih.gov/aam/mouse/inbred-mice-1, http://research.jax.org/grs/type/inbred/). In addition, it is generally accepted that there are no significant changes in genome configuration, primarily in regard to the number and position information of nucleotides, during development, differentiation, and/or in response to environmental stressors of an individual mouse or human [30]. In this experiment, we investigated whether the configuration of the germ line genome of an individual inbred mouse diversifies during development, differentiation, and/or in response to environmental stressors, creating a pool of spatially divergent somatic genomes. Genome-wide MLV-ERV landscapes of a set of 18 different somatic organs (13 nonlymphoid and 5 lymphoid) and sperm collected from a single male C57BL/6J inbred mouse (12 weeks old) were analyzed to examine spatial genomic variations within an individual. We acknowledge that the somatic tissues, which were subjected to the MLV-ERV landscaping analysis, have residual blood cells. However, we anticipate that the genomic DNA derived from the blood cells represents a minute fraction of the DNA preparations from the individual somatic tissues. As such, it is unlikely that the residual blood cells significantly affect the genome-wide MLV-ERV landscape patterns of the somatic tissues. The MLV-ERV landscapes of the 13 nonlymphoid organs were highly variable and were also different from the sperm pattern, although the profile of about a dozen visible I-PCR amplicons was shared among all of them (Figure 2(a)). It is likely that variable landscape patterns of the 13 somatic tissues, in comparison to the germ line cells (sperm), are closely linked to the temporal and spatial activities of MLV-ERVs during the various developmental stages of the organs. In addition, an examination of a group of five lymphoid organs (bone marrow, thymus, spleen, mesenteric lymph node, and inguinal lymph node) demonstrated marked variations in the I-PCR amplicon patterns (Figure 2(b)). It is expected that genomic configurations of lymphoid organs (both primary and secondary) are different from germ line cells due to rearrangements during lymphoid cell development/differentiation. Overall, some organs had more visible I-PCR amplicons than the others, and no unique banding pattern of I-PCR amplicons, which can differentiate the genomes of somatic organs from germ line genomes, was identified. These findings indicate that genome-wide MLV-ERV landscapes of a single C57BL/6J mouse are spatially diversified from the germ line configuration depending on organ type (and potentially cell type), creating a pool of somatic genome variants.

### 3.2. Polymorphic MLV-ERV Landscapes in Various Brain Compartments of C57BL/6J Inbred Mice

**Figure 3:**
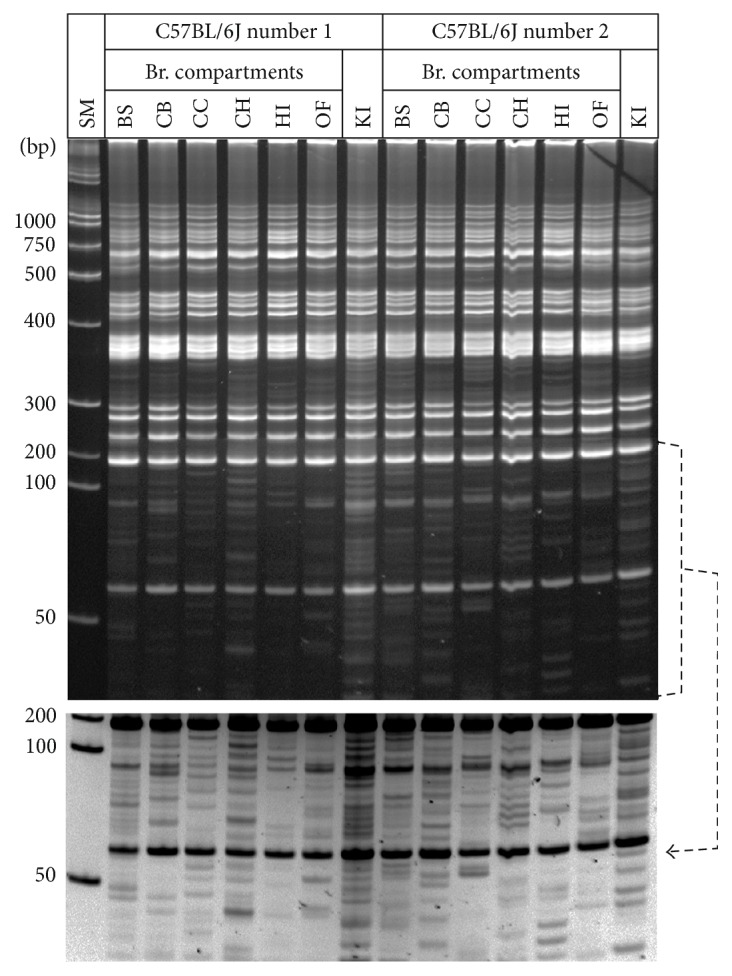
Variable MLV-ERV landscapes in different brain compartments of C57BL/6J inbred mice. Variations in the I-PCR amplicon banding patterns were visible among the six different brain compartments of two female C57BL/6J mice. In addition, within each compartment, the landscape patterns were not shared by the two mice. A reversed image of the selected area is presented for better resolution. SM: size marker; BS: brain stem; CB: cerebral cortex; CC: corpus callosum; CH: cerebellar hemisphere; HI: hippocampus; OF: olfactory bulb; KI: kidney.

To examine whether there are spatial variations in MLV-ERV landscapes of the brain, genomic DNA isolated from six different brain compartments (brain stem, cerebral cortex, corpus callosum, cerebellar hemisphere, hippocampus, and olfactory bulb) of two C57BL/6J female mice (5 weeks old) were subjected to the I-PCR landscaping analysis (Figure 3()). There were noticeable variations in the I-PCR amplicon patterns among the six brain compartments, and within each compartment, the MLV-ERV landscape patterns were not shared by the two mice examined. It is likely that the I-PCR amplicon patterns obtained from this experiment are not specific for the individual brain compartments. Instead, each pattern represents temporal and spatial variations in the MLV-ERV landscapes in the genomes of a plethora of different cell types and/or cells, at varying stages of their life span, in the brains of these inbred mice.

### 3.3. Temporal Variations in MLV-ERV Landscapes in the Primary and Secondary Immune Organs of C57BL/6J Inbred Mice

**Figure 4:**
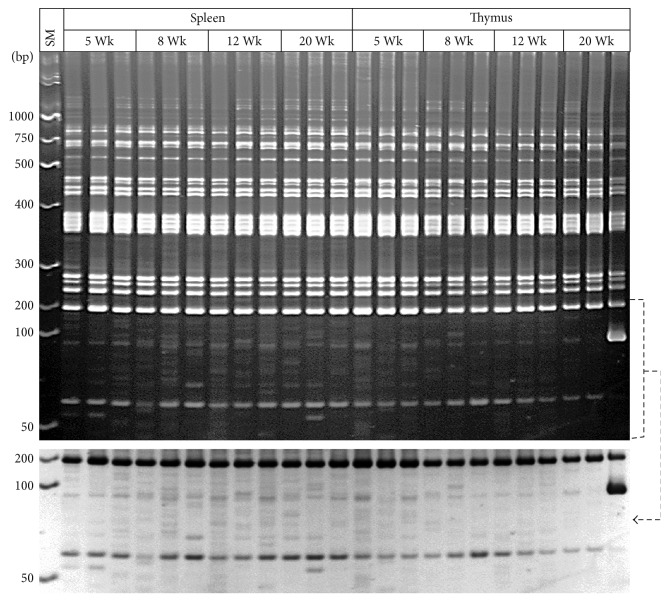
Variations in MLV-ERV landscapes in the immune organs (primary—thymus and secondary—spleen) of C57BL/6J inbred mice of different ages. Substantial variations in the MLV-ERV landscapes were observed in the thymus and spleen genomic DNA among all four age groups (5, 8, 12, and 20 weeks) of the C57BL/6J male mice. No age-specific or immune organ-specific I-PCR amplicon banding patterns were identified. For better resolution, a reversed image of the selected area is shown. Wk: week; SM: size marker.

The cells in immune organs, such as thymus (primary) and spleen (secondary), constantly respond to a wide range of intrinsic and external stressors, some of which may have the potential for stimulating MLV-ERV activity. We examined whether MLV-ERV landscapes of the thymus and spleen are temporally altered and whether specific patterns can be identified in the four age groups (5, 8, 12, and 20 weeks) of C57BL/6J male mice. Variations were observed in the MLV-ERV landscapes in both types of immune organs among all four age groups of mice; however, no age group-specific or immune organ-specific I-PCR amplicon patterns were discernible (Figure 4()). Interestingly, the thymic MLV-ERV landscapes of the third mouse of the 20 weeks old group contained one unusually strong I-PCR amplicon band for which a follow-up investigation would be warranted. It is likely that an additional set of landscaping probes may be needed to collect higher-resolution datasets which allow for potential identification of age group- and/or immune organ-specific MLV-ERV landscapes.

### 3.4. Variations in MLV-ERV Landscapes of Spatially Separated Organ Sets in a Single C57BL/6J Inbred Mouse

**Figure 5:**
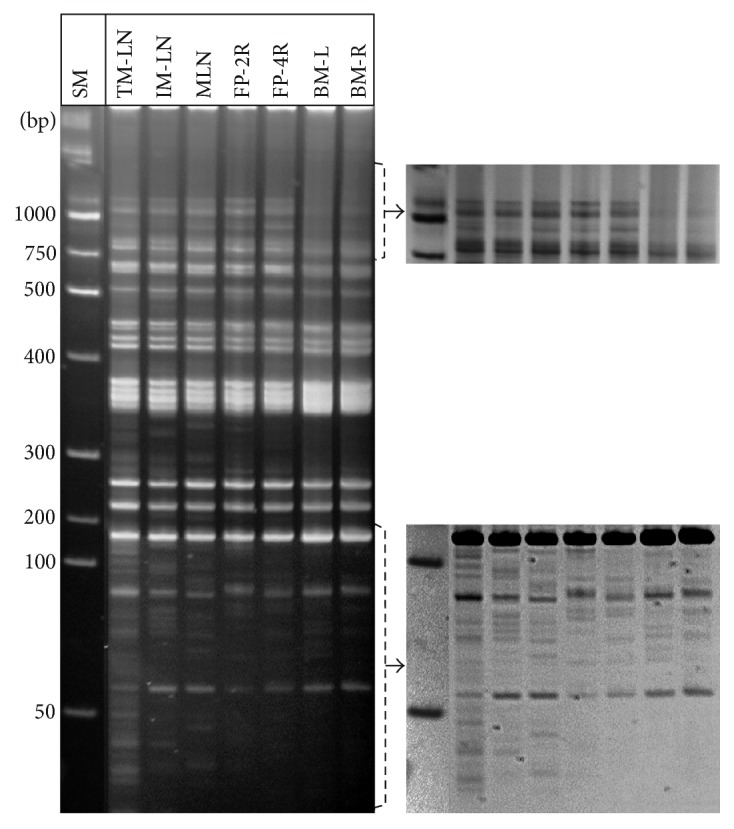
Polymorphic MLV-ERV landscapes of spatially separated organ sets of a C57BL/6J mouse. There were considerable variations in MLV-ERV landscapes among the individual sets of three lymph nodes (thoracic mammary, inguinal mammary, and mesenteric) and two mammary fat pads (number 2 right and number 4 right) derived from a female C57BL/6J mouse. In contrast, bone marrow samples isolated from the left and right femurs shared I-PCR amplicon patterns which are visibly different (density and banding pattern) from the landscapes of lymph nodes and mammary fat pads. A selected area is shown as a reversed image for better analysis. SM: size marker; TM-LN: thoracic mammary lymph node; IM-LN: inguinal mammary lymph node; FP: fat pad; L: left; R: right; BM: bone marrow.

Certain types of organs (e.g., lymph nodes and mammary glands) in humans and mice are found in more than one location (e.g., left side and right side). In this experiment, we examined variations in MLV-ERV landscapes in a set of three lymph nodes (thoracic mammary, inguinal mammary, and mesenteric), a pair of mammary fat pads (number 2 right and number 4 right), and a pair of bone marrow samples (derived from left and right femurs) isolated from a 27-month-old C57BL/6J female mouse. Examination of I-PCR amplicon profiles revealed substantial variations in MLV-ERV landscapes among the individual sets of three lymph nodes and two mammary fat pads (Figure 5). In comparison to the lymph node and mammary fat pad sets, the two bone marrow samples isolated from left and right femurs had somewhat similar I-PCR amplicon patterns. Furthermore, it was interesting to observe that MLV-ERV landscapes of the two bone marrow samples have unique I-PCR amplicon profiles with significantly lower band densities compared to the lymph nodes and mammary fat pads. The differences in overall density of the MLV-ERV landscapes can be attributed to the fact that the population of cells in the bone marrow (primary lymphoid organ) is considered to be less differentiated compared to the cells in the lymph nodes (secondary lymphoid organ). In fact, the cells in the lymph nodes, which are constantly subjected to a host of stressors, are expected to maintain elevated levels of MLV-ERV activity, leading to an increase in the number of MLV-ERV positions/copy numbers in the affected genomes. The findings from this study demonstrate spatial variations in MLV-ERV landscapes among the same organ types at different locations in an inbred C57BL/6J mouse.

### 3.5. Individual-, Gender-, and Organ Type-Specific Variations in MLV-ERV Landscapes in the Kidney and Liver of C57BL/6J Inbred Mice

**Figure 6:**
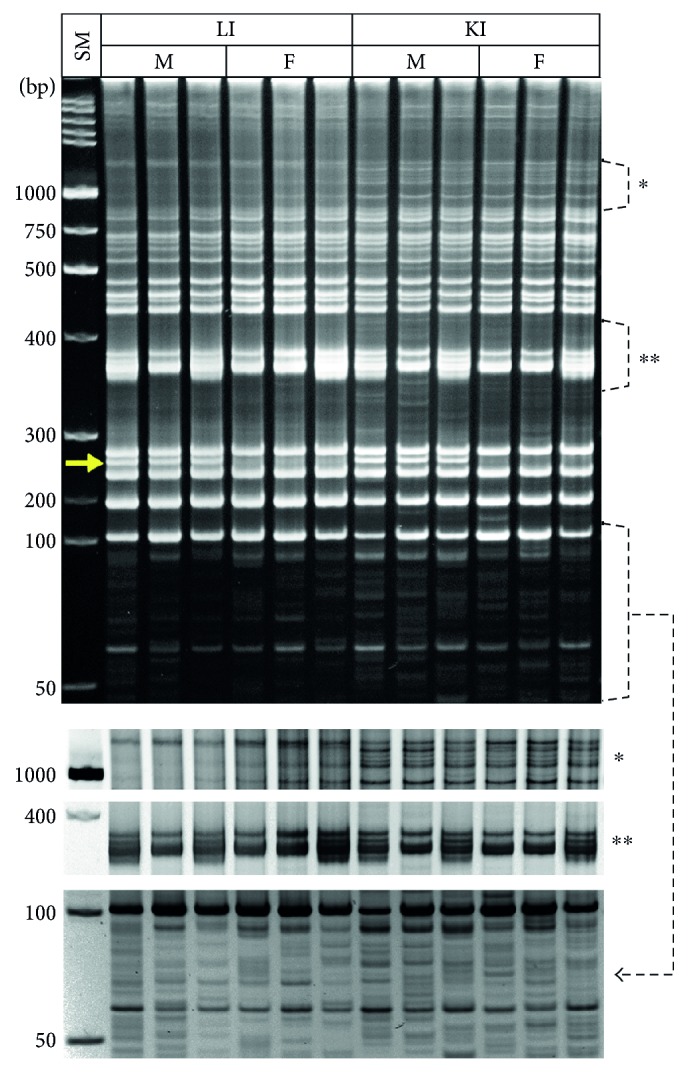
Variable MLV-ERV landscapes in C57BL/6J inbred mice depending on gender, individual, and organ type. There were apparent variations in MLV-ERV landscapes of the kidney and liver genomes between females and males of C57BL/6J mice; these variations include one distinct I-PCR amplicon band (indicated with an arrow) found only in male mice. In addition, MLV-ERV landscapes of the liver genomes lack a set of amplicon bands in contrast to the kidney genomes (∗). Furthermore, each mouse (female or male) had either one of the two distinct sets of I-PCR amplicon bands, regardless of gender (∗∗). A reversed image of a selected area (indicated with an arrowed dotted line) is also presented. SM: size marker; KI: kidney; LI: liver; F: female; M: male.

It has been reported that chromosome Y of C57BL/6J male mice is densely populated with a plethora of repetitive elements [24]. In this experiment, we examined the differences in MLV-ERV landscapes between three females and three males using the kidney and liver samples from 19-month-old C57BL/6J mice. Among other variations, there was one distinct I-PCR amplicon band which is found only in male mice in both tissues (Figure 6); thus, the male-specific amplicon band is presumably derived from chromosome Y. A close examination of the banding patterns revealed that MLV-ERV landscapes of the liver genomes from all six mice (three females and three males) lack a cluster of amplicon bands which are clearly present in all six kidney genomes (Figure 6^∗^). Unexpectedly, two distinct clusters of I-PCR amplicon bands were identified in the MLV-ERV landscapes of both kidney and liver genomes. Interestingly, each of the six mice had either one, but not both, of the two cluster patterns in its MLV-ERV landscape, regardless of its gender identity (Figure 6^∗∗^). This finding provides another piece of solid evidence that genomic configuration with regard to the MLV-ERV landscape is variable depending on the individual in the population of C57BL/6J inbred mice of the same gender.

### 3.6. Organ Type-Specific Variations in Genomic Copy Number of MLV-ERVs between the Kidney and Liver of C57BL/6J Inbred Mice

**Figure 7:**
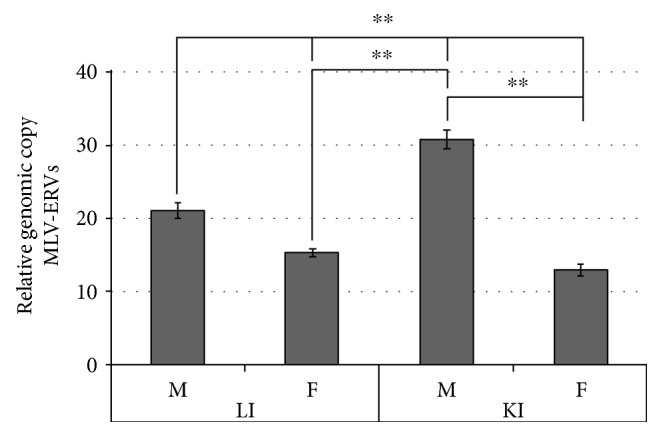
Gender- and organ type-dependent differences in genomic copy number of MLV-ERVs in C57BL/6J inbred mice. There were higher copy numbers of MLV-ERVs in the kidney and liver genomes of the same male mice, which are described in Figure 6, in comparison to female mice. Importantly, within all three male mice, but not in females, the kidney genomes had substantially more MLV-ERV copies compared to the liver genomes. ^∗∗^*p* ≤ 0.01.

To quantify differences in the genomic copy number of MLV-ERVs between the three female and three male C57BL/6J inbred mice which were analyzed in the previous section, real-time PCR analysis was performed using genomic DNA isolated from the kidneys and livers. It is expected that the kidney and liver tissues, which were subjected to the genomic MLV-ERV copy number analysis, have residual blood cells. Since the genomic DNA derived from the residual blood cells is only a very small fraction of each tissue's total DNA preparation, it is unlikely that the blood cells affect the analysis of genomic MLV-ERV copy numbers between the two tissues. As somewhat expected based on the properties of chromosome Y, male mice had higher copy numbers of MLV-ERVs in the kidney and liver genomes compared to female mice (Figure 7()). Unexpectedly, we found that within the three male C57BL/6J mice, the kidney genomes have substantially more MLV-ERV copies compared to the liver genomes whereas no significant differences between the two organs were found in female mice. This finding coincides with previous reports that genomic copy numbers of porcine endogenous retroviruses (PERVs) are variable among different organs of a single pig [31–33]. We acknowledge that it is critical to carefully design and execute any ERV (e.g., MLV-ERV and PERV) copy number study since the results can vary depending on a number of experimental conditions, such as DNA quality and PCR parameters. In consideration of the relatively advanced age (19 months) of the mice examined in this study, it would be interesting to repeat the same experiment with a series of age groups from neonates to over two years old. A future investigation which focuses on mapping putative additional MLV-ERV loci in the kidney genomes, in comparison to the liver genomes, of these male C57BL/6J mice may provide a novel insight into the kidney biology of mouse and human and potentially aging.

## 4. Discussion

It is critical to clearly define and confirm the genetic constancy of animals which are employed in a wide range of experimental models for studying gene function, toxicity of candidate compounds, diseases, and others [34, 35]. The definition and degree of the genetic constancy of research animals are tunable depending on the specific aim(s) of individual studies. For example, when a candidate compound for therapeutic drug development is evaluated for its side effects and/or toxicities, the genetic constancy of the experimental animals does not have to be highly stringent. Conversely, for studies which investigate the function of genes using a pair of defective (conventional mutant or engineered) and its matching control animals, one must rely on stringent genetic constancy in order to accomplish a proper evaluation and translation of the experimental outcomes.

It is not uncommon to encounter variations in morphologic phenotypes among a population of a specific inbred mouse strain, such as C57BL/6J ([36], https://www.jax.org/news-and-insights/1995/october/microphthalmia-and-ocular-infections-in-inbred-c57-black-mice, personal communication). Some of these phenotypic variations within an inbred mouse population are explained primarily by irreversible genetic drift events due to the genetic fixation of accumulated mutations (typically in conventional genes) which are often discovered serendipitously or as outcomes of troubleshooting experiments [37]. It was suggested that the current repertoire of gene SNPs and other DNA markers (e.g., microsatellite elements) is not sufficient for screening genetic drift in mice [37]. To circumvent the detrimental effects of cumulative genetic drifts over time, producers of research mice have implemented control programs, such as the Genetic Stability Program (Jackson Laboratory) and the Genetic Monitoring Program (Taconic Biosciences). One of the key shared features of these programs is the cryopreservation of embryos for future replacement of foundation mice. Jackson Laboratory reported that “Inbred strains within this program effectively remain genetically unchanged for at least the period of the program (projected 25 years)” [37].

As described in the Introduction, not even a single chromosome in the NCBI's reference mouse genome, which is derived from C57BL/6J inbred mice, has yet been completely sequenced ([1], http://www.ncbi.nlm.nih.gov/assembly/GCF_000001635.24/). In addition, the NCBI's reference mouse chromosome Y, which was estimated to be ~16 Mb in length up until early 2013, is now annotated to contain ~92 Mb in it. These findings summarize the inherent difficulties in understanding genome/chromosome biology as well as decoding/sequencing the entire genetic information system of humans and animals. In addition to the estimated ~20,000 conventional genes annotated in the reference mouse genome, the vast majority of the mouse genome is occupied by a plethora of TREome members. In contrast to conventional genes, the TREome is highly diverse within the mouse as well as human populations ([38–40], unpublished). Moreover, it has been well-documented that some members of the TREome have *gene* sequences which code for functional proteins, such as the superantigen of mouse mammary tumor virus-type ERVs and human endogenous retroviruses [41–43]. Importantly, certain TREome members of mice and humans respond to a range of stressors, leading to an increase in their activity [44, 45]. Acquired TREome (e.g., MLV-ERVs) activities during the life course of an inbred mouse could involve the following critical processes: (1) DNA-dependent RNA polymerization, resulting in TREome gene transcripts, (2) protein synthesis from the TREome gene transcripts, (3) RNA-dependent DNA polymerization (reverse transcription) in the cytoplasm to make DNA copies of the TREome transcripts, (4) virion (e.g., MLV-ERV) assembly, and (5) random integration of DNA copies of the TREome transcripts into the genome. Apparently, one of the critical impacts imposed by the accumulation of the acquired TREome activities would be exemplified by individual-specific dynamic (temporal and spatial) alterations in the TREome landscapes of the affected genomes.

One can argue that the entirety, or at least the majority, of the information embedded in the dynamic genomes are relevant in determining phenotypic details in humans, mice, and other species. Moreover, the acquired activity of MLV-ERVs, as well as other TREs, would play a critical role in the dynamic (temporal and spatial) structural shaping of the genomes of each inbred mouse strain. As such, surveillance of the acquired activity of the diverse TREome, as demonstrated in this study, would provide critical and valuable information for understanding the relationships between the complex and dynamic genetic characteristics and phenotypes within a single strain as well as among different strains of laboratory mice. We suggest that a mouse genetics surveillance system be established for laboratory mice which accounts for the inherent diversity and acquired activity of the TREome and interrogates temporal and spatial variations in genomic landscapes. This TREome-based genetics surveillance system would serve as a synergistic tool for the current monitoring systems (e.g., the Genetic Stability Program and the Genetic Monitoring Program) which primarily rely on the cryopreservation of embryos and survey for the polymorphisms of specific genes and microsatellites despite the absence of a single complete reference genome. Successful development of the TREome-based mouse genetics surveillance system would be applied to high-resolution genetic identification and monitoring of a broad range of species, from humans to plants, which all harbor their own dynamic TREomes.

## Supplementary Material

Supplementary Table 1. PCR primers and reaction conditions.
