# Supplementary material for: Highly Variable Genomic Landscape of Endogenous Retroviruses in the C57BL/6J Inbred Strain, Depending on Individual Mouse, Gender, Organ Type, and Organ Location
Source: Int J Genomics. 2017 Aug 29;2017:3152410. doi: 10.1155/2017/3152410 (PMC5603323; doi:10.1155/2017/3152410)
Supplement: Supplementary file 1 — Supplementary Table 1. PCR primers and reaction conditions. [file 3152410.f1.docx]

| **Primer** | **Sequence** | **PCR Conditions** | | | |
| --- | --- | --- | --- | --- | --- |
|  |  | Denaturation | Annealing | Elongation | Cycles |
| ISL-F1 | 5'-GACTGAGTCGCCCGGGTA-3' | 94°C / 30 sec | 58°C / 60 sec | 72°C / 120 sec | 32 |
| ISL-R1 | 5'-GCGGTTGAGAATACAGGGTC-3' |  |  |  |  |
| ERV-U1 | 5'-CGGGCGACTCAGTCTATCGG-3' | 95°C / 30 sec | 55°C / 60 sec | 72°C / 60 sec | 40 |
| ERV-U2 | 5'-CAGTATCACCAACTCAAATC-3' |  |  |  |  |
